# Dendrimer-mediated delivery of *N*-acetyl cysteine to microglia in a mouse model of Rett syndrome

**DOI:** 10.1186/s12974-017-1004-5

**Published:** 2017-12-19

**Authors:** Elizabeth Nance, Siva P. Kambhampati, Elizabeth S. Smith, Zhi Zhang, Fan Zhang, Sarabdeep Singh, Michael V. Johnston, Kannan Rangaramanujam, Mary E. Blue, Sujatha Kannan

**Affiliations:** 1 Department of Anesthesiology and Critical Care Medicine, Johns Hopkins University School of Medicine, Baltimore, MD 21205 USA; 2 Center for Nanomedicine, Department of Ophthalmology, Wilmer Eye Institute, Johns Hopkins University School of Medicine, Baltimore, MD 21231 USA; 3 Department of Chemical and Biomolecular Engineering, Johns Hopkins University, Baltimore, MD 21218 USA; 4 Department of Materials Science and Engineering, Johns Hopkins University, Baltimore, MD 21218 USA; 5 Hugo W. Moser Research Institute, Kennedy Krieger, Inc., Baltimore, MD 21205 USA; 6 Present address: Department of Chemical Engineering, University of Washington, Seattle, WA 98105 USA

**Keywords:** Rett syndrome, Microglia, System Xc^−^, *N*-Acetyl cysteine, PAMAM dendrimer, *Mecp2*-null, Glutamate

## Abstract

**Background:**

Rett syndrome (RTT) is a pervasive developmental disorder that is progressive and has no effective cure. Immune dysregulation, oxidative stress, and excess glutamate in the brain mediated by glial dysfunction have been implicated in the pathogenesis and worsening of symptoms of RTT. In this study, we investigated a new nanotherapeutic approach to target glia for attenuation of brain inflammation/injury both in vitro and in vivo using a *Mecp2*-null mouse model of Rett syndrome.

**Methods:**

To determine whether inflammation and immune dysregulation were potential targets for dendrimer-based therapeutics in RTT, we assessed the immune response of primary glial cells from *Mecp2*-null and wild-type (WT) mice to LPS. Using dendrimers that intrinsically target *activated* microglia and astrocytes, we studied *N*-acetyl cysteine (NAC) and dendrimer-conjugated *N*-acetyl cysteine (D-NAC) effects on inflammatory cytokines by PCR and multiplex assay in WT vs *Mecp2*-null glia. Since the cysteine-glutamate antiporter (Xc^−^) is upregulated in *Mecp2*-null glia when compared to WT, the role of Xc^−^ in the uptake of NAC and l-cysteine into the cell was compared to that of D-NAC using BV2 cells in vitro. We then assessed the ability of D-NAC given systemically twice weekly to *Mecp2*-null mice to improve behavioral phenotype and lifespan.

**Results:**

We demonstrated that the mixed glia derived from *Mecp2*-null mice have an exaggerated inflammatory and oxidative stress response to LPS stimulation when compared to WT glia. Expression of Xc^−^ was significantly upregulated in the *Mecp2*-null glia when compared to WT and was further increased in the presence of LPS stimulation. Unlike NAC, D-NAC bypasses the Xc^−^ for cell uptake, increasing intracellular GSH levels while preventing extracellular glutamate release and excitotoxicity. Systemically administered dendrimers were localized in microglia in *Mecp2*-null mice, but not in age-matched WT littermates. Treatment with D-NAC significantly improved behavioral outcomes in *Mecp2*-null mice, but not survival.

**Conclusions:**

These results suggest that delivery of drugs using dendrimer nanodevices offers a potential strategy for targeting glia and modulating oxidative stress and immune responses in RTT.

**Electronic supplementary material:**

The online version of this article (10.1186/s12974-017-1004-5) contains supplementary material, which is available to authorized users.

## Background

Rett syndrome (RTT) is an X-linked disorder that affects 1:10,000 female births and results in motor dysfunction, autonomic irregularities including abnormal breathing, and intellectual disability [1–6]. In 95% of cases of RTT, the gene that encodes for methyl-CpG-binding protein 2 (*MECP2*) is mutated [1–6]. *MeCP2* is a ubiquitous transcription repressor and activator protein [7] that is expressed in neurons and glial cells. However, the mechanism(s) by which a loss of *MeCP2* drives the pathology in RTT is not clearly defined. Immune dysregulation with a shift in the Th1/Th2 balance has been demonstrated in peripheral circulation of patients with RTT [8]. This was shown to be associated with redox imbalance, increased oxidative stress, and decreased glutathione (GSH) in patients with RTT [8]. Both the aberrant cytokine signaling and the redox imbalance appear to modulate the phenotype severity in patients with RTT [8]. *MeCP2* plays an integral role in the regulation of microglia response to inflammatory stimuli [9]. Glial dysfunction has been implicated in the pathogenesis and worsening of symptoms in RTT [10, 11]. An increase in reactive oxidative species and glutamate levels [8, 10, 12–14], both of which can induce or amplify inflammatory signaling, has been observed in some patients with RTT and in the brains of mouse models of RTT. There is also encouraging evidence that improving microglial function could decrease or even arrest injury and promote repair and regeneration [9, 11, 15]. However, approaches that rescue *Mecp2* expression in microglia have not shown efficacy in mouse models of RTT [16, 17].

*N*-Acetyl cysteine (NAC), a precursor for GSH formation, has both anti-oxidant and anti-inflammatory properties and is widely used clinically in children and adults [18–21]. Inflammation causes depletion of GSH in astrocytes and microglia and decreases their ability to maintain redox balance and prevent oxidative injury [22, 23]. NAC has shown limited efficacy in treating irritability in children with autism spectrum disorders (ASDs) [20]. NAC is typically administered in very high doses (up to 800 mg per day), because of the poor bioavailability due to protein binding of the –SH groups [24, 25]. Cysteine, the rate-limiting amino acid for GSH synthesis, is transported intracellularly as a dimer via the system cysteine-glutamate antiporter (Xc^−^) that exchanges one glutamate for one cysteine [26–28]. The light-chain subunit Xc^−^ is primarily responsible for its antiporter action and is upregulated in the presence of inflammation and oxidative stress. The system Xc^−^ is largely responsible for maintaining the extracellular glutamate concentration in the brain by exporting glutamate outside the cell while importing cysteine to maintain intracellular GSH levels. In this way, NAC, a precursor of cysteine, can increase intracellular GSH and thereby improve oxidative injury. Therefore, upregulation of system Xc^−^ in various disorders may be a protective mechanism for increasing the anti-oxidant defense within the cell by increasing GSH synthesis. However, l-cysteine, the active component in NAC, has reported neurotoxic effects due to over activation of *N*-methyl-d-aspartate (NMDA) receptors on neurons [29], and administration of NAC may potentially lead to increased extracellular glutamate locally in the brain, worsening the injury. Upregulation of system Xc^−^ also has been implicated in glutamate-induced injury of neurons, and inhibitors of system Xc^−^ are currently being explored for decreasing glutamate toxicity in various indications [28, 30, 31]. Hence, delivery of NAC selectively to glial cells bypassing the Xc^−^ transporter may help improve oxidative injury while decreasing neuronal toxicity.

We have previously shown that in the presence of neuroinflammation, generation-4 hydroxyl-terminated poly(amidoamine) (G4-OH PAMAM) dendrimers (~ 4 nm) cross the blood-brain barrier (BBB) and accumulate selectively in activated microglia and astrocytes in the brain of newborn rabbits with cerebral palsy (CP), but not in age-matched healthy controls [32]. These same dendrimers accumulate in activated glia in a canine model of hypothermic circulatory arrest-induced brain injury [33] and in a mouse model of ischemia-induced neonatal white matter injury [34]. The goal of this study was to determine whether methyl-CpG-binding protein 2 mouse gene (*Mecp2*)-null glia respond differently to an inflammatory stimulus when compared to wild-type (WT) glia, and if dendrimer-conjugated *N*-acetyl cysteine (D-NAC) is effective in attenuating these responses in the KO and WT glia. We also evaluated whether D-NAC bypasses the Xc^−^ transporter to deliver NAC and cysteine intracellularly which would improve intracellular glutathione levels without increasing extracellular glutamate. As a translational application of this concept, we also explored the potential of this glial cell-directed therapy using D-NAC administered *after* symptom onset, for improvement in neurological outcomes in a clinically relevant mouse model of RTT.

## Methods

### Synthesis and characterization of D-Cy5 and D-NAC conjugates

Fluorescently labeled dendrimers (D-Cy5) were synthesized and characterized using a previously reported procedure [35]. Briefly, in the first step, bifunctional dendrimers were synthesized using Fluorenylmethyloxycarbonyl chloride (FMOC) protection/deprotection chemistry resulting in six to eight primary amines (–NH_2_) on the dendrimer surface. In the second step, the –NH_2_ groups on the dendrimer surface was reacted with *N*-hydroxysuccinimide monoester-Cy5 dye to obtain the D-Cy5 conjugates by NHS-NH_2_ click chemistry. The product was purified using dialysis and the D-Cy5 was characterized using ^1^H NMR for Cy5 loading and HPLC to assess the purity of the conjugate.

D-NAC conjugates were synthesized by following the multistep synthetic procedure previously established and reported by our lab [33]. Briefly, in the first step, G4-OH dendrimers were partially functionalized using 4-aminobutyric acid, resulting in bifunctional dendrimers with ~ 18–20 NH_2_ groups on the surface using tert-butyloxycarbonyl (BOC) protection/deprotection chemistry. Further, in the second step, the bifunctional dendrimers were reacted with succinimidyl-3-(2-pyridyldithio)propionate (SPDP) in the presence of N,N-Diisopropylethylamine as a base with anhydrous dimethylformamide (DMF) as solvent under N_2_ atmosphere for 6 h followed by the addition of *N*-acetyl-l-cysteine in situ and were stirred at room temperature for another 6 h. The conjugates were purified via dialysis (in DMF for 24 h followed by water dialysis for 6 h) and gel permeation chromatography (GPC) to obtain D-NAC conjugates. As part of our standard authentication process, D-NAC conjugates were characterized using ^1^H NMR and HPLC to assess the drug loading and purity, as reported previously [33]. D-NAC stability and drug release kinetics were evaluated in 80% human pooled plasma with intracellular (500 μM) and extracellular (10 μM) GSH levels. A concentration of 3 mg/mL was maintained at 37 °C in a water bath with constant shaking. At various time points, 400 μL of plasma was sampled, plasma proteins were protonated and precipitated using 400 μL of − 20 °C-cold 0.05% methanolic TCA, and the solution was sonicated for 5 min and centrifuged at 10,000 rpm for 5 min at 4 °C. The supernatant was collected, and methanol was evaporated under nitrogen flush, then it was reconstituted in 400 μL of water and subjected to HPLC analysis (reported previously) [32] to evaluate drug release.

### Cell culture and in vitro treatments

#### Primary mixed glia from WT/*Mecp2*-null mice brains

Both hemispheres of the cerebral cortex from 1-week-old WT and *Mecp2*-null mice were excised under sterile conditions, and their meninges were removed carefully by submerging the cerebral cortex in sterile warm phosphate-buffered saline (PBS). The cleaned cortices were suspended in 0.5% trypsin for 15 min to digest the tissue. The trypsin was neutralized using Dulbecco’s modified Eagle’s medium (DMEM; 4.5 g, glucose 10% FBS and 1% Penn/Strep) medium (Cellgro, Manassas, VA, USA). The tissue was minced using constant pipetting, and the tissue suspension was filtered through a 40-μm nylon cell strainer (BD Falcon, Franklin Lakes, NJ, USA) to remove tissue debris. The filtered cell suspension was centrifuged at 1000 rpm for 5 min at 4 °C, and the pellet was resuspended in culture media and was seeded in collagen-coated T-175 flasks and left undisturbed for enhancing their attachment of the cells for a period of 7 days at 37 °C and 5% CO_2_ atmosphere. Later, the cell debris and unattached cells in the culture were removed by gently changing the media every 2 days to grow and expand until 90% confluency was achieved for another week.

#### Cytotoxicity analysis of D-NAC in primary mixed glia

Mixed glial cells were seeded in a 96-well plate (Costar, Cambridge, MA, USA) and incubated at 37 °C for 48 h in their respective growth medium. After 48 h, the cells were treated with a medium containing different concentrations (10–200 μg) of free NAC, D-NAC (containing equivalent NAC), and G4-OH PAMAM (10–200 μg) for 24 h. The cells treated with a fresh medium serve as control. Cell viability was assessed by using an MTT cell proliferation assay kit (Molecular Probes; Invitrogen, OR, USA). Absorbance was read at 540 nm using the fluorescence microplate reader (BioTek Instruments, Winooski, VT, USA), and cell viability was determined as percent absorbance relative to untreated control cells.

#### Lipopolysaccharide activation of primary mixed glia and D-NAC efficacy studies

The mixed glial cells were seeded in 24-well plates and allowed to attach for 48 h. After 48 h, the cells were activated with 100 ng/mL lipopolysaccharide (LPS) (from *Escherichia coli* O127:B8) (Sigma-Aldrich, St. Louis, MO, USA) for 6 h by following previously established protocols [36, 37]. Following LPS activation, the cells were treated with D-NAC (10 and 100 μg/mL on NAC basis) and free NAC (10 and 100 μg/mL) for 6 h. We chose a 6-h treatment window based on our previous in vitro studies where < 90% of cells uptake dendrimers [36–38] and in vivo experiments that have shown robust uptake of dendrimers in activated microglial cells [35, 39]. Also, the results from this experiment reflect the efficacy of D-NAC or free NAC uptake over a limited period of 6 h which would mimic a clinical or in vivo scenario following systemic administration. After 6 h of treatment, the cells were washed gently with warm PBS and replenished with medium containing 100 ng/mL LPS for 24 h to mimic sustained neuroinflammation and to investigate how the treated cells behave under continuous inflammatory stress [36]. After 24 h, the medium was collected and stored at − 80 °C for nitric oxide (NO) and multiplex cytokine ELISA analysis (Meso Scale Discovery, Rockville, MD, USA). Experimental scheme is provided in Fig. 2, left panel. Each experimental condition had *n* = 4.

#### NO assay for evaluating the anti-oxidant activity of D-NAC in primary glial cells

Anti-oxidant activity of D-NAC treatment on primary mixed glial cells was evaluated by measuring NO released in the medium using a Griess reagent detection kit (Promega, Madison, WI, USA). Briefly, the frozen medium samples of the primary mixed glial cells treated with D-NAC and free NAC were thawed on ice and briefly centrifuged at 1000 rpm at 4 °C for 5 min. The supernatant was diluted appropriately, and the samples (50 μL each) were placed in 96-well glass bottom, black-walled plates (Corning, NY, USA) and reacted with sulfanilamide and NED solutions provided in the kit. The end point of the reaction (color change, according to the kit’s protocol) was measured using a plate reader at 520 nm with a correction at 550 nm. The NO levels were calculated using the calibration graph equation as per the manufacturer’s instructions.

#### Multiplex cytokine ELISA assay

A multiplex cytokine ELISA panel (Meso Scale Discovery, Rockville, MD, USA) was used to quantify TNF-α, IL-1β, IL-2, IL-4, IL-5, IL-6, IL-10, IL-12p70, INF-γ, and C-X-C motif chemokine 1 (CXCL1). As per the manufacturer’s instructions, standards and medium samples (50 μL) were incubated in the plate for 2 h. After washing, the plate was incubated for 2 h in a detection antibody solution for all 10 analytes. The plate was washed again three times, read buffer was pipetted into all wells, and then the plate was placed on a QuickPlex SQ 120 plate reader (Meso Scale Discovery, Rockville, MD, USA) and fluorescence from all 10 analytes was measured. Upon analyses of all 10 analytes, we found that the majority of samples had IL-2, IL-4, and IL-5 concentrations below detection range and therefore have not been included in the results.

#### RNA extraction and RT-PCR analysis of cytokines in primary mixed glial cells

The cell pellets were snap frozen in liquid nitrogen and stored in − 80 °C until analysis. Total RNA was extracted with 500 μL of TRIzol reagent (Life Technologies, Grand Island, NY, USA) according to the manufacturer’s instruction, and the extracted RNA was purified using ethanol. Two micrograms for total RNA was reverse transcribed to complementary DNA (cDNA) using a high-capacity cDNA reverse transcription system (Life Technologies) according to the manufacturer’s instructions. qRT-PCR was performed with Fast SYBR Green Master Mix (Life Technologies) by a StepOnePlus Real-Time PCR System (Life Technologies) on GAPDH (forward: TGTCGTGGAGTCTACTGGTGTCTTC; reverse: CGTGGTTCACACCCATCACAA) and Xc^−^ (forward: CTTCGATACAAACGCCCAGATA; reverse: CTGAATGGGTCCGAGTAAAGAG). The expression of GAPDH messenger RNA (mRNA) was used to normalize the expression levels of target genes and was calculated by the comparative cycle threshold Ct method (2^−ΔΔCt^). Data is expressed as fold change from wild type.

#### Murine BV2 cell culture

BV2 cells were used for the subsequent studies related to Xc^−^ inhibition. Since this experimental paradigm involved using a cysteine-free medium and application of an Xc^−^ inhibitor, the hardier BV2 cells were used for these studies instead of primary glial cells. The murine BV2 cell line passage 21 (P 21) was cultured in DMEM (Life Technologies, Grand Island, NY, USA) (phenol red free) supplemented with 10% heat-inactivated fetal bovine serum (Hi-FBS; Invitrogen Corp., Carlsbad, CA, USA) and 1% antibiotics (penicillin/streptomycin) (Invitrogen Corp., Carlsbad, CA, USA).

#### Anti-inflammatory assay in BV2 cells

BV2 cells were seeded at a concentration of 1 × 10^6^ cells per well in a 12-well plate. Cells were activated with LPS (*E. coli* serotype O127:B8; Sigma-Aldrich, St. Louis, MO, USA) at a concentration of 100 ng/mL for 3 h. After 3 h, the cells were treated with 1 mL of the medium containing 0.1, 1, 10, and 100 μg/mL concentrations of NAC and D-NAC (containing equivalent concentrations of NAC conjugated to dendrimers) for a period of 12 h. After 12 h, the medium was removed, and the cells were washed gently with a warm medium and replenished with a new medium containing 100 ng/mL of LPS for a period of 24 h. The cell culture medium was sampled at the end of 24 h, and TNF-α levels were measured using a mouse TNF-α ELISA kit (R&D Systems, Minneapolis, MN, USA). The effect of LPS treatment on Xc^−^ expression was determined by evaluating the mRNA expression of Xc^−^ normalized to the housekeeping gene GAPDH in cells exposed to LPS or medium. These studies were done to confirm that Xc^−^ is upregulated in BV2 cells following LPS stimulation (similar to the Rett and WT glia) and to determine the optimal doses of D-NAC, based on TNF-α attenuation, to be used for the subsequent studies.

#### In vitro glutamate and glutathione assays after Xc^−^ inhibition in BV2 cells

After LPS pre-treatment (1 × 10^6^ cells per well in a 12-well plate), the BV2 cells were treated with 100 μM sulfasalazine (Sigma, St. Louis, MO, USA) to inhibit the system Xc^−^. The sulfasalazine concentration is chosen based on previous studies by Chung et al. [40] and Sleire et al. [41]. Before efficacy experiments, a pilot study was done to test different concentrations of sulfasalazine (500, 200, 100, and 10 μM) for cytotoxicity. Concentrations of 100 μM and lower did not induce cell death (> 90% cell viability) while 200 μM and above had < 70% viability. After sulfasalazine pre-treatment, the cells were treated with 1 mL of the medium (DMEM without l-glutamine, sodium pyruvate, and cysteine) (Gibco, Life Technologies, Grand Island, NY, USA) containing 100 μg of cysteine, free NAC, or D-NAC (equivalent concentration of conjugated NAC) along with 50 μM of sulfasalazine for 8 h. After 8 h, the medium was collected, and the cells were gently washed with warm sterile PBS and replaced with a fresh phenol red-free DMEM medium for 18 h. The medium collected at 8 h was diluted twice using a phenol red-free medium, and glutamate levels were detected using an Amplex^®^ Red glutamic acid/glutamate oxidase assay kit (Molecular Probes; Life Technologies, Grand Island, NY, USA). The end point was measured using a fluorescence microplate reader (BioTek Instruments, Winooski, VT, USA) using excitation at 560 mm and emission at 590 nm. The glutamate levels were calculated using the calibration graph according to the manufacturer’s instructions. To quantify intracellular GSH levels, cells at the treatment end point (after 18 h) were subjected to trypsin treatment and centrifugation (2000 rpm for 5 min at 4 °C), and then cell pellets were washed with PBS. Cell pellets were frozen immediately in liquid nitrogen. To lyse the cells, the cell pellets were thawed on ice and treated with ice-cold lysis buffer and the cells were homogenized using constant pipetting followed by centrifugation for 10 min at 4 °C, at 5000 rpm. The supernatant was collected, and total GSH levels were detected using a glutathione detection assay kit (Abcam, Cambridge, UK). The end point was measured using a fluorescence plate reader at 360 nm emission/460 nm excitation, and the total GSH (GSH + GSSG) was calculated using the calibration graph according to the manufacturer’s instructions. The total GSH levels were normalized using total protein levels in the sample using a BCA assay kit (Thermo Fisher, Halethorpe, MD, USA).

### Mouse model of RTT (*Mecp2*^tm1.1Bird^)

All procedures were approved by the Johns Hopkins University Animal Care and Use Committee. *Mecp2*-heterozygous (HET) mice were bred with WT C57/BL6 males to produce WT and HET female and WT and *Mecp2*-null male mice. Due to poor outcomes with breeding of the HET mice, an alternative breeding strategy was adopted. WT male and female mice were placed in a cage together, and 6–7 days later, a HET female was added. After 10–11 days, the male was removed and the WT female and *Mecp2*-heterozygous female were left in the cage together. Typically, the WT dam gave birth about a week before the HET dam. WT litters were culled to three pups 2–3 days after birth; the rest were euthanized when pups from HET dams had toes clipped between postnatal days 5 and 7 for genotyping and animal identification. Pups were kept with the dam until PD 28 when they were weaned. Single-sex mice were housed together (no more than five mice per cage). Mice were genotyped using a PCR protocol provided by the Jackson Laboratory and as specified in our previous publications [42, 43].

#### Immunohistochemistry and image analysis for biodistribution of D-Cy5

*Mecp2*-null mice and normal healthy age-matched WT mice were administered 55 mg/kg of D-Cy5 intraperitoneally (i.p.) at 1 week of age (pre-symptomatic) and 7 weeks of age (when the animals are most symptomatic and have increased mortality beyond this age). Animals were euthanized 24 h after D-Cy5 administration and perfused with normal saline. Brain sections were stained for microglia/macrophages and astrocytes with rabbit anti-Iba1 (microglia) or rabbit anti-GFAP (astrocytes) and then donkey anti-rabbit IgG Alexa 594 or chicken anti-rabbit IgY Alexa 488. Maximum intensity-projected, confocal z-stack images (20–30 μm thick) were obtained using a Zeiss LSM 710 microscope to detect co-localization of D-Cy5 with Iba1+ cells or to assess microglia morphology. While multiple regions of the forebrain were scanned, the region photographed in Fig. 5 was located just above the corpus callosum near the midline.

#### D-NAC therapy for in vivo efficacy study

Beginning on PD 21, *Mecp2*-null and WT mice received i.p. injections of (1) D-NAC (10 mg/kg on a NAC basis), (2) PBS, or (3) NAC (10 mg/kg) twice weekly (every 3–4 days, Monday and Friday, after behavioral assessments). This dosage was chosen based on extensive previous work demonstrating that this dosage is effective in reducing neuroinflammation and oxidative stress as well as improving behavioral deficits in various animal models including neonatal hypoxic ischemia and maternal inflammation model of cerebral palsy [32, 34, 44]. Furthermore, as this is a chronic disease, we chose to redose twice per week as we know that maximal uptake and release of D-NAC occurs within 24–48 h after administration and the dendrimer would be completely eliminated from the body by that time [35]. We hypothesized that the released NAC will be utilized, and in order to replenish the therapeutic concentration of NAC in the brain, we chose to administer twice weekly to maximize the efficacy of D-NAC and compared the results with free NAC injected twice a week at the same concentration. Animals were euthanized when 10% body weight was lost within a 1-week time period or when neurobehavioral scores exceeded a composite score of 18.

#### Neurobehavioral analysis in *Mecp2*-null mice

*Mecp2*-null mice treated with D-NAC, NAC, or PBS and WT mice underwent neurobehavioral analyses twice per week, prior to treatment. Weight measurements (g) were taken prior to any behavioral assessment on each treatment day. Behavioral assessments included appearance, mobility, gait, tremor (whole body), respiratory irregularities, paw clenching, paw clench time (if clenching was present), and paw wringing. All behavioral tests have been previously reported and validated in the literature [45]. Assessment of appearance included whether the mouse was emaciated or bloated, if fur was groomed, the buildup of debris or discharge around the eyes, loss of eyesight, the presence of cataracts, and the presence of a hunched posture. All other behavioral tests were scored on a scale of 0 to 3, with 0 being normal and 3 being severe (see Additional file 1 for scoring scheme).

Animals were placed in an open field for 1 min to acclimate. Animals were video recorded for 1 min in the open to observe mobility, gait, and the presence of tremor. Animals were then suspended in the air by the tail for 10 s, repeated three times, to note any paw clenching, and if paw clenching was present, the number of paws that were clenched as well as the length of time and pattern to the clenching were noted. Animals were then placed in an open flat palm of the hand to observe respiratory irregularities and presence of tremors. The composite behavior score was added and reported for comparison among the different treatments.

### Statistics

Using Prism GraphPad, *t*-tests (mRNA) and one- and two-way ANOVAs (NO, cytokine ELISA data) were conducted. A *p* value less than or equal to 0.05 was considered statistically significant. When conducting post hoc *t*-tests, the *p* value was adjusted for multiple comparisons using a Bonferroni correction or Sidak multiple comparisons adjustment. For in vivo survival data, a Kaplan-Meier survival curve was implemented. Linear mixed models that accounted for a variation among repeated measurements over time within litters were used to estimate the differences between composite behavioral scores in four groups (*Mecp2*-null mice treated with D-NAC, NAC, or PBS, and WT male mice) and across time. These analyses were performed using the R version 3.2.2 (R Foundation for Statistical Computing, Vienna, Austria).

## Results

### D-Cy5 and D-NAC conjugates

To evaluate the biodistribution and localization of intravenously injected dendrimers, we labeled the dendrimers with Cy5, a near-IR dye, which avoids tissue autofluorescence. Bifunctional dendrimer was synthesized with minimal –NH_2_ groups (six to seven) on the dendrimer surface enabling Cy5 to be conjugated via a NH_2_-NHS click reaction. ^1^H NMR spectrum analysis indicated that ~ 1.2 molecules of Cy5 were conjugated to each dendrimer.

HPLC characterization demonstrated that the D-Cy5 conjugates are pure [with an elution time of 14.8 min (at 645 nm)] and completely different from G4-OH [14.4 min (at 205 nm)] (data not shown). The D-Cy5 conjugates were stable and intact in human pooled plasma for 48 h at 37 °C. D-NAC conjugates were designed to avoid pre-mature release and to enable release of NAC under intracellular conditions in target cells. We utilized disulfide linkage (–S–S–) to conjugate NAC molecules to the dendrimer surface, which can be cleaved at intracellular glutathione levels. Partially aminated G4-OH (bifunctional) dendrimers were synthesized by conjugating ~ 23–25 molecules of 4-(*tert*-butoxycarbonylamino)butyric acid (GABA-BOC) in the first step. The BOC groups were deprotected using trifluoroacetic acid (TFA) in anhydrous dichloromethane (DCM) (1:3) for 6 h, and the solvent was evaporated to remove excess TFA and side products to obtain bifunctional dendrimers with 20–22 –NH_2_ groups on the dendrimer surface. In the third step, SPDP was conjugated to the surface of –NH_2_ groups via an amide bond. In the final step, NAC was conjugated using a disulfide bond to the dendrimers. The structure of D-NAC was established by the ^1^H NMR spectrum (Additional file 2). Characteristic multiplet peaks at 1.64 ppm corresponded to methylene (–CH_2_) of GABA linker. A singlet peak at 1.84 ppm corresponded to acetyl (–C_2_H_3_O), and multiplet peaks at 4.46 ppm corresponded to methyl (–CH) of NAC confirming successful conjugation of NAC to the dendrimer. Additionally, the appearance of a new peak at 4.0 ppm corresponded to modified methylene (–CH_2_) protons of dendrimer. Using a proton integration technique, we estimated that ~ 21 NAC molecules were conjugated to each dendrimer molecule. In the HPLC chromatogram (Additional file 2), the appearance of a new peak at 19.65 min was different from that of free NAC peak (6.14 min) and G4-OH peak (14.9 min) (data not shown), confirming the formation of product. We did not observe any characteristic peaks of free NAC and dendrimer peak, suggesting the D-NAC conjugate was pure (~ 95%). The size and surface charge of G4-OH dendrimers were 4.4 ± 0.2 nm and 4.5 ± 0.2 mV, respectively. Conjugating ~ 20 molecules of GABA linker resulted in increased surface charge (12.8 ± 0.2 mV) due to primary –NH_2_ groups on dendrimer surface. After conjugating NAC to the dendrimer surface, the size and zeta potential for D-NAC conjugates were 5.2 ± 0.1 nm and 8.9 ± 0.2 mV, respectively (Additional file 2). The increase in size and surface charge does not affect the targeting ability and biodistribution of dendrimer as recently reported by our group [44].

### Stability and drug release characteristics of D-NAC

We previously studied the stability and drug release characteristics of D-NAC conjugates in PBS [32]. At extracellular GSH concentrations (2 μM) at 37 °C, D-NAC conjugates were stable and did not release any detectable NAC (in any forms: NAC linker, NAC-NAC, NAC-GSH) over 24 h. At physiological conditions (PBS 7.4, 37 °C; data not shown) and in plasma (37 °C), the D-NAC conjugate was stable and did not release NAC over a 24-h period (Additional file 3). However, at intracellular GSH concentrations (250 μM), the conjugate released the drug readily within 3.5 h (Additional file 3), indicating that the use of a disulfide linker enables rapid release of NAC from the conjugate only when it is exposed to an intracellular GSH-rich environment [46, 47]. Interestingly, D-NAC was stable in human plasma (with 10 μM GSH) at 37 °C over a period of 24 h, releasing less than 3% of NAC (Additional file 3).

### Xc^−^ expression was upregulated in *Mecp2*-null mice

**Fig. 1:**
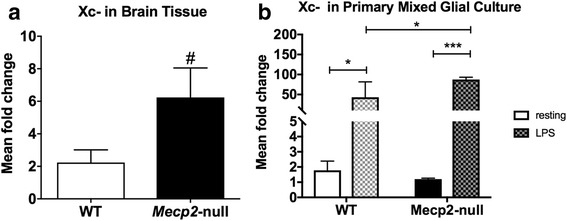
XCT expression in wild-type (WT) versus *Mecp2*-null mice. **a** At 1 week of age, XCT mRNA expression was increased in *Mecp2*-null mice. **b** XCT mRNA expression increased significantly after stimulation with lipopolysaccharide toxin (LPS; checkered bars) in primary mixed glial cultures from WT and *Mecp2*-null mice. The response to LPS was also significantly increased in *Mecp2*-null compared to WT primary mixed glial cells. Mean values ± SEM for graphs in **a** and **b** (^#^ *p* = 0.06; *p < 0.05; ***p < 0.001)

To determine if *Mecp2* deletion led to changes in Xc^−^ expression, we evaluated Xc^−^ (the gene) mRNA expression in the brains of *Mecp2*-null mice and WT mice by PCR. Xc^−^ mRNA had a trend of an increase at 1 week of age (*t*(13) = 2.302, *p* < 0.06; Fig. 1a). We also examined Xc^−^ mRNA expression in primary mixed glia culture from the *Mecp2*-null and WT mouse cortex under resting conditions and when LPS was used to activate the cells (Fig. 1b). There was a trend for an effect of genotype [*F*(1,11) = 3.049, *p* = 0.1], a significant effect of treatment [*F*(1,11) = 37.34, *p* < 0.0001], and a trend for an interaction of state/treatment and genotype [*F*(1,11) = 3.19, *p* = 0.1]. Specifically, *Mecp2*-null cells had a significant increase in Xc^−^ mRNA expression from the rest to LPS stimulation [*t*(11) = 5.33, *p* < 0.001] as did WT cells [*t*(11) = 3.183, *p* < 0.05]. *Mecp2*-null cells treated with LPS also showed a significant increase in Xc^−^ expression compared to WT cells with LPS stimulation [*t*(11) = 2.6, *p* = 0.048].

### Immune dysregulation in *Mecp2*-null primary mixed glial culture

#### G4-OH PAMAM and D-NAC were not cytotoxic to primary mixed glial cells

We and others have previously published that G4-OH PAMAM demonstrates minimal toxicity in vivo up to 500 mg/kg [32, 48, 49] and with no cytotoxicity in cell cultures observed up to concentrations of 500 μg/mL [36, 50]. Before proceeding to efficacy experiments, we evaluated the cytotoxicity profile for G4-OH PAMAM, D-NAC, and free NAC in *Mecp2*-null primary mixed glial cells to verify the biosafety of the treatment concentrations. Both G4-OH PAMAM and D-NAC at a concentration of 200 μg/mL did not cause any cytotoxicity to primary glial cells. Approximately 94.4 ± 1.76 and 88.9 ± 3.0% of cells were viable after G4-OH PAMAM and D-NAC treatment, respectively, whereas free NAC treatment at 200 μg/mL caused ~ 20.0% cell death (Additional file 4). At concentrations below 200 μg/mL (100 and 10 μg), the cell viability was greater than 95%; hence, we used 100 μg/mL for D-NAC and free NAC as the highest concentration for in vitro efficacy studies.

**Fig. 2:**
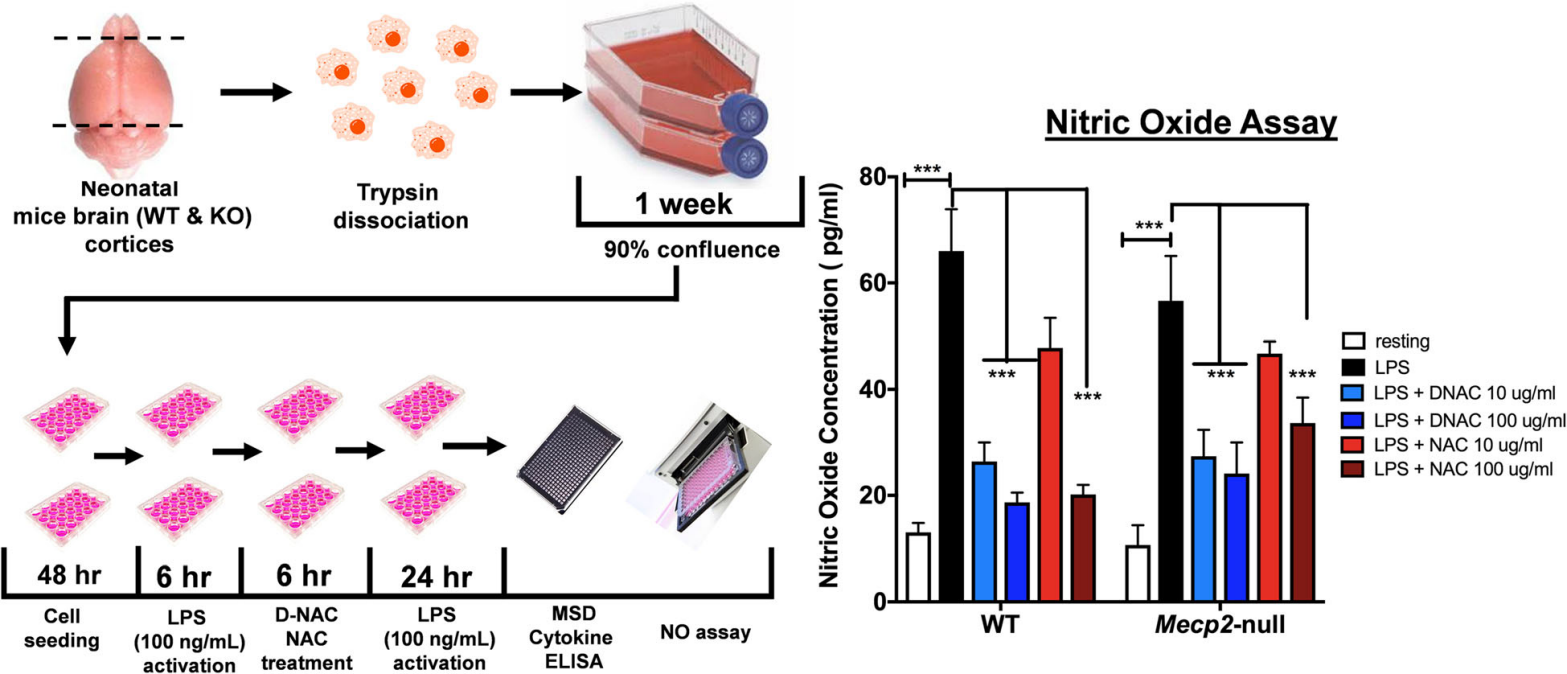
Increased nitric oxide (NO) release from *Mecp2*-null primary mixed glial cells challenged with LPS. Cortical tissue samples from the brains of 1-week-old *Mecp2*-null and WT mice were taken, and cells were dissociated and plated to grow. Once confluent, cells were plated, activated with LPS, and treated with D-NAC and NAC (left panel). Both WT and *Mecp2*-null glial cells showed equivalent NO release in response to LPS (black bars) that was diminished significantly by D-NAC (blue bars) administration (right panel). Only the high dose of free NAC was effective in decreasing the response to LPS (red bars)

Both hemispheres of the cerebral cortex from 1-week-old WT and *Mecp2*-null mice were used to perform mixed glial cell experiments. The schematic in the left panel of Fig. 2 illustrates the experimental flow and the outcome measures.

### Nitric oxide production in *Mecp2*-null mixed glial culture

The potential anti-oxidant activities of D-NAC and free NAC treatment on primary mixed glial cells were evaluated using Griess reagent detection of NO released into a medium under varying conditions. No difference in NO production was observed in WT and *Mecp2*-null glial cells under resting conditions [*t*(6) = 1.15, *p* > 0.2]. LPS exposure led to a significant increase in NO production to a similar extent in both WT and *Mecp2*-null glia. However, D-NAC treatment at 10 and 100 μg/mL concentrations significantly decreased NO production in both WT and treated *Mecp2*-null glial cells exposed to LPS stimulation [WT D-NAC 10 μg/mL: *t*(30) = 5.64, *Mecp2*-null D-NAC 10 μg/mL: *t*(30) = 4.728, *p* < 0.001; WT D-NAC 100 μg/mL: *t*(30) = 4.72, *Mecp2*-null D-NAC 100 μg/mL: *t*(30) = 4.253, *p* < 0.001], whereas only the 100 μg/mL dose of NAC was effective in decreasing the level of NO release [WT: *t*(30) = 5.47, *Mecp2*-null: *t*(30) = 43.34, *p* < 0.001; Fig. 2, right panel].

### *Mecp2*-null glia exhibited a dysregulated cytokine profile under resting conditions and an exaggerated response to LPS stimulation

**Fig. 3:**
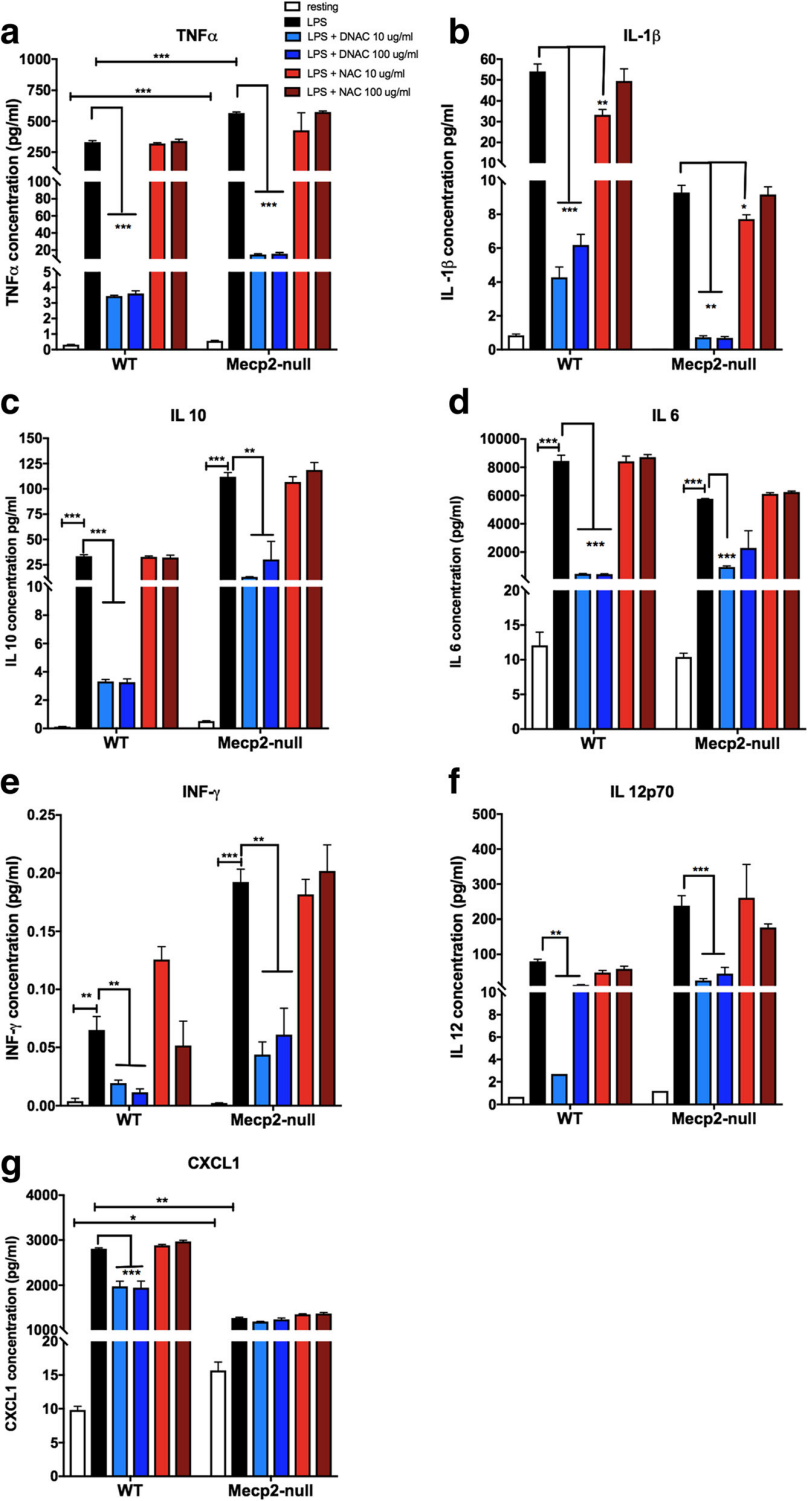
Increased cytokine release in WT and *Mecp2*-null primary mixed glial cell cells challenged with LPS. A multiplex ELISA was conducted on the media collected from the mixed glial cell cultures. At rest (white bars), TNF-α (**a**), IL-10 (**c**), and CXCL1 (**g**) release was increased in *Mecp2*-null mixed glial culture compared to WT whereas release of IL-1β (**b**) was decreased. No group differences in IL-6, INF-γ, and IL-12 were observed at rest (**d**–**f**). After LPS stimulation (black bars), concentrations of TNF-α (**a**), IL-10 (**c**), INF-γ (**e**), and IL-12 (**f**) were significantly higher in *Mecp2*-null compared to WT glial cells. In contrast, blunting of the response to LPS stimulation for IL-1β (**b**), IL-6 (**d**), and CXCL1 (**g**) was observed in *Mecp2*-null glial cells compared to WT. Across the board, D-NAC 10 μg/mL and D-NAC 100 μg/mL (blue bars) consistently lowered all cytokine levels significantly in WT and *Mecp2*-null LPS-treated cells (all *p* ≤ 0.001) with the exception of the chemokine CXCL1 in *Mecp2*-null glial cells. In most cases, free NAC (red bars) was not effective except for IL-1β in which 10 μg/mL NAC decreased IL-1b levels significantly (*p < 0.05; **p < 0.01; ***p < 0.001)

Cytokine levels were measured using a multiplex ELISA assay on the culture supernatant collected at the end of the experiment. Under resting conditions, the supernatant from *Mecp2*-null glia had significantly higher levels of TNF-α [*t*(6) = 7.44, *p* < 0.001; Fig. 3a], IL-10 [*t*(5) = 8.60, *p* < 0.001; Fig. 3c], and CXCL1 [*t*(6) = 4.328, *p* < 0.01; Fig. 3g] but significantly lower levels of IL-1β [*t*(5) = 12.47, *p* < 0.001; Fig. 3b] compared to WT cells. No group differences in IL-6, INF-γ, and IL-12 were observed at rest (Fig. 3d–f; all *p* > 0.2). LPS exposure resulted in a disproportionate response of the inflammatory cytokines in *Mecp2*-null glial cells after LPS administration. Concentrations of TNF-α, IL-10, INF-γ, and IL-12 were significantly higher in *Mecp2*-null compared to WT glial cells [TNF-α: *t*(6) = 15.39; IL-10: *t*(6) = 17.43; INF-γ: *t*(6) = 7.88; IL-12: *t*(6) = 9.905; all *p* < 0.001]. In contrast, blunting of the response to LPS stimulation for IL-1β, IL-6, and CXCL1 was observed in *Mecp2*-null glial cells compared to WT [IL-1β: *t*(6) = 12.4; IL-6: *t*(6) = 6.53; CXCL1: *t*(6) = 60.16; all *p* < 0.001]. The directional relationships of these findings have been summarized in Table 1.

**Table 1:** Comparison of cytokine release of *Mecp2*-null glial to wild-type glial cells

Cytokine	Baseline (resting)	After LPS stimulation
TNF-α	↑	↑
IL-1β	↓	↓
IL-10	↑	↑
IL-6	–	↓
INF-γ	–	↑
IL-12p70	–	↑
CXCL1	↑	↓

↑ increase from WT; ↓ decrease from WT; – no difference from WT

### Treatment with D-NAC was more effective than NAC in attenuating the cytokine response after LPS exposure

When evaluating the impact of treatment, we observed a significant effect of treatment for all cytokines in WT and *Mecp2*-null glial cells [TNF-α: *F*(4,29) = 47.43; IL-1β: *F*(4,28) = 62.58; IL-10: *F*(4,28) = 46.36; IL-6: *F*(4,29) = 120.8; INF-γ: *F*(4,27) = 6.347; IL-12: *F*(4,24) = 8.192; CXCL1: *F*(4,29) = 40.52, all *p* < 0.001]. Across the board, D-NAC 10 μg/mL and D-NAC 100 μg/mL consistently lowered all cytokine levels significantly compared to WT or *Mecp2*-null LPS-treated cells (all *p* ≤ 0.001) with the exception of the chemokine CXCL1 in *Mecp2*-null glial cells. Free NAC was not effective in most cases even at the highest dose, except for IL-1β in which 10 μg/mL NAC decreased IL-1b levels (*p* < 0.05).

#### LPS exposure leads to increased Xc^−^ expression in BV2 cells

**Fig. 4:**
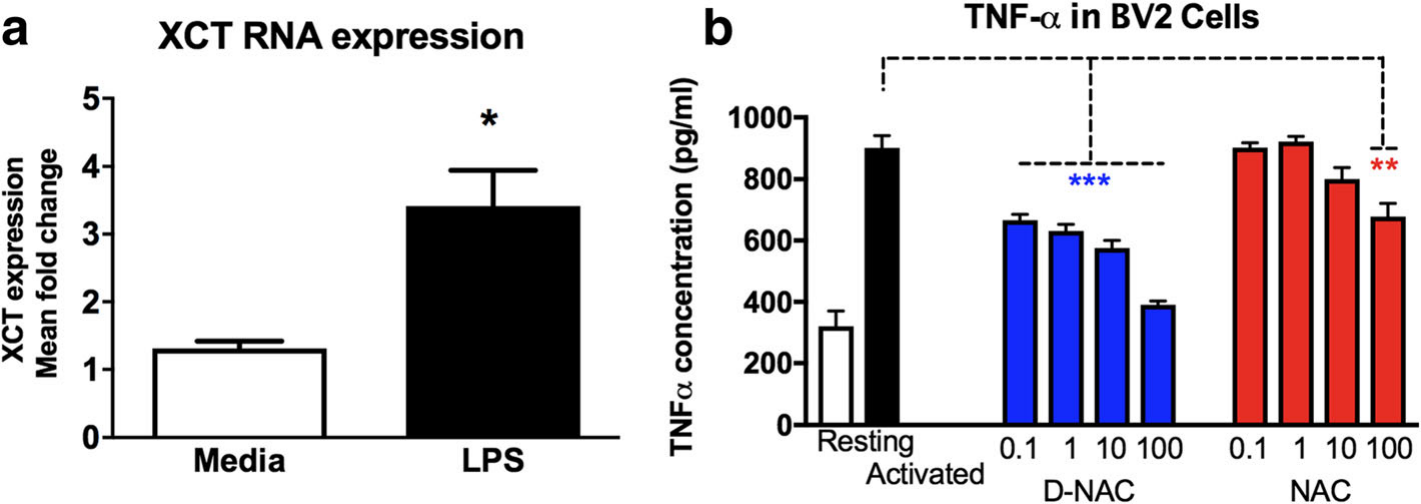
Effects of LPS stimulation on XCT and TNF-α expression. **a** XCT expression in BV2 cells was increased after LPS stimulation (*p < 0.05). **b** BV2 cell cultures treated with D-NAC showed dose-dependent decreases in TNF-α concentration in the supernatant as compared to LPS-treated (activated) cultures. Only the highest dose of NAC (100 μg/mL) was effective at reducing TNF-α concentration

Mouse BV2 cells were activated using LPS, as described in our past studies [37, 51]. We used mouse BV2 cells for the studies evaluating Xc^−^ since these cells can be cultured in glutamine- and cysteine-free media. We have previously shown that LPS exposure to BV2 cells leads to a pro-inflammatory response (increase in TNF-α release) and a depletion of GSH levels without cell death [36]. Here, we investigated the effect of LPS pre-treatment on Xc^−^ expression. Exposure to LPS resulted in a significant upregulation of mRNA expression of Xc^−^ in the BV2 cells [*t*(28) = 2.564, *p* = 0.016; Fig. 4a].

#### D-NAC is more effective than NAC in suppressing TNF-α in BV2 cells

Exposure to LPS resulted in a ~ 10-fold increase in the TNF-α release by BV2 cells (Fig. 4b). After stimulating with LPS, BV2 cells were incubated with 1 mL of the medium containing 0.1, 1, 10, and 100 μg/mL concentrations of free NAC or D-NAC for 12 h followed by 24 h of LPS-containing medium. Thus, anti-inflammatory activity by NAC or D-NAC was reflected by a decrease in TNF-α concentration in the supernatant. Both D-NAC and NAC showed dose-responsive suppression of TNF-α levels in the supernatant [D-NAC: *F*(4,15) = 52.74, *p* < 0.0001; NAC: *F*(4, 15) = 9.873, all *p* < 0.001; Fig. 4b], whereas free NAC was only effective at reducing TNF-α at the highest concentration (*p* < 0.01; Fig. 4b). D-NAC at a dose of 0.1 or 1 μg/mL was as effective as 100 μg/mL of free NAC in decreasing TNF-α levels. Our group has previously reported that dendrimers are rapidly taken up by cells in vitro [52]. Significant reduction of TNF-α in D-NAC-treated cells can be attributed to improved intracellular availability of NAC via dendrimers. Moreover, it has to be taken into account that the cells were exposed to both NAC and D-NAC only for 6 h and the suppression of TNF-α reflects to an amount of NAC or D-NAC internalized by the cells in that limited period.

#### l-Cysteine and NAC-mediated increase in intracellular GSH is dependent on Xc^−^ for transport into the cell while D-NAC bypasses system Xc^−^

**Fig. 5:**
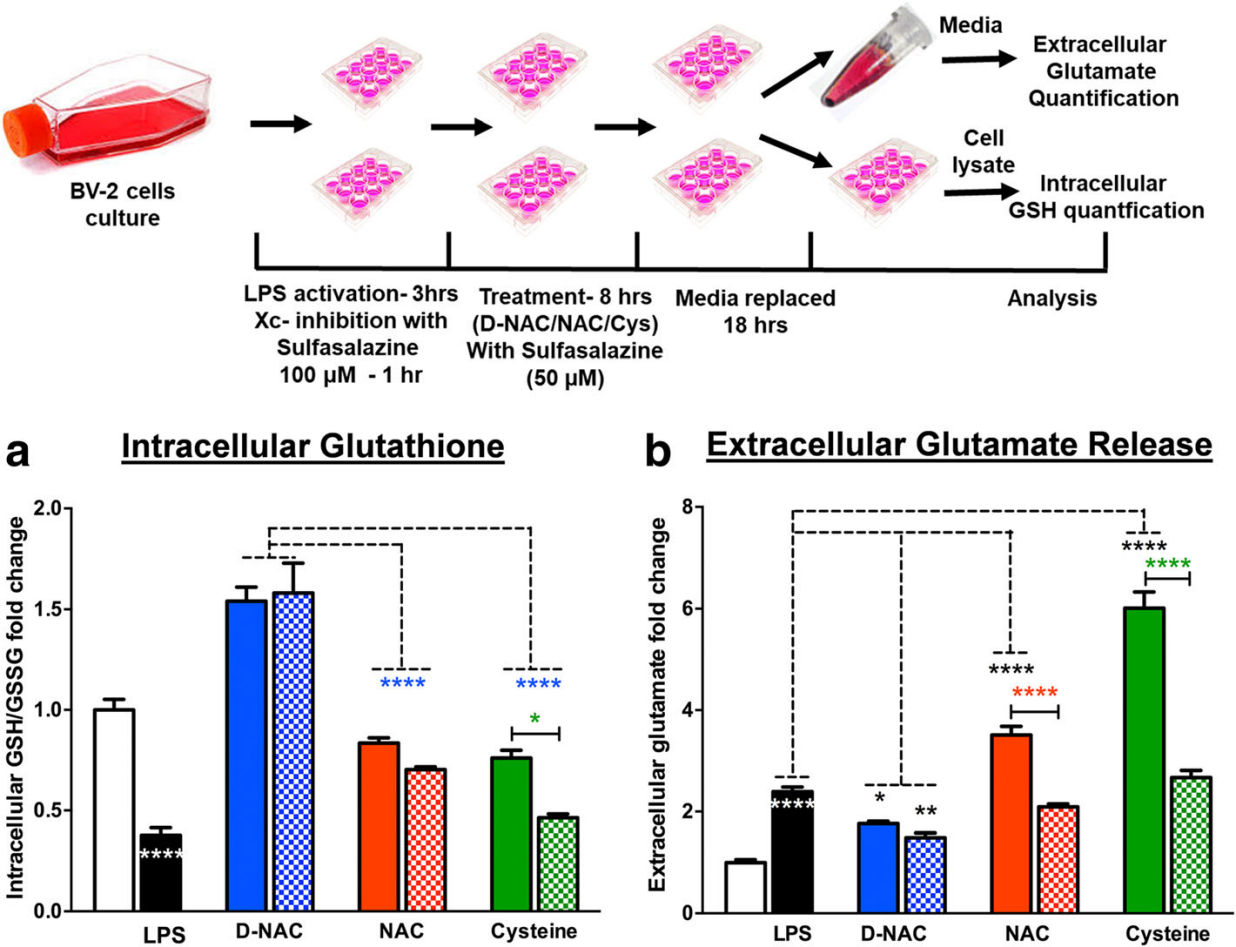
Role of Xc^−^ in NAC internalization in BV2 cells. *Mecp2*-null and WT brains were acquired, and cells were dissociated and plated to grow. Once confluent, cells were reseeded and treated with LPS (3 h) and sulfasalazine (1 h) and then incubated in cysteine, NAC, or D-NAC with sulfasalazine for 8 h. Then, the cells were incubated in fresh media for 18 h. Cells and media were then taken and used to assess intracellular glutathione levels and extracellular glutamate release. To assess the mechanism of internalization of D-NAC as compared to NAC and cysteine, extracellular glutamate and intracellular glutathione levels were assessed in LPS-activated BV2 cells after treatment when Xc^−^ was functional (without sulfasalazine (solid bars)) and when it was blocked with an Xc^−^ inhibitor (with sulfasalazine (cross-hatched bars)). **a** In D-NAC-treated cells, GSH increased regardless of Xc^−^ functionality/blockade (blue bars), suggesting that D-NAC bypasses Xc^−^ to exert its anti-oxidant effect intracellularly. In NAC- and cysteine-treated cells, Xc^−^ blockade resulted in a decrease in GSH production, suggesting that NAC and cysteine were internalized in large part via Xc^−^. **b** Glutamate levels increased with LPS administration. When Xc^−^ was functional, NAC (red bars) and cysteine (green bars) showed increased glutamate levels, but when Xc^−^ was blocked, glutamate release decreased in NAC- and cysteine-treated samples. In contrast, regardless of Xc^−^ functionality/blockade, D-NAC (blue bars) was effective in reducing glutamate release (*p < 0.05; **p < 0.01; ***p < 0.001; white asterisks denote the comparison between resting and LPS conditions; the black asterisks denote the comparison between LPS-stimulated and LPS + D-NC, NAC, or cysteine conditions; the blue asterisks denote the comparison between LPS + D-NAC and LPS + NAC and LPS + cysteine conditions; the red and green asterisks denote the comparison between LPS + NAC and LPS + NAC + sulfasalazine and LPS + cysteine and LPS + cysteine + sulfasalazine)

To determine whether l-cysteine, NAC, and D-NAC were dependent on system Xc^−^ for intracellular transport and increasing glutathione levels, LPS-activated BV2 cells were treated with l-cysteine, NAC, or D-NAC (all at 100 μg/mL of cysteine or NAC basis) in the presence or absence of sulfasalazine, a potent Xc^−^ inhibitor (50 μM that is not toxic to BV2 cells; the schematic is shown in Fig. 5 top panel). Activation of the BV2 cells with LPS led to a 50% reduction in baseline levels of GSH (*p* < 0.0001; Fig. 5b) and ~ 2.5-fold higher levels of extracellular glutamate (Fig. 5a). Although treatment with l-cysteine and NAC led to an increase in intracellular GSH levels, treatment with D-NAC at the same dose (on a NAC basis) was significantly better [*F*(2,21) = 54.75, *p* < 0.0001; Fig. 5a]. Treatment with NAC and l-cysteine was associated with an increase in extracellular glutamate [*F*(4,72) = 11.28, *p* < 0.001] that was not seen with D-NAC treatment. Extracellular glutamate levels were lower in D-NAC and D-NAC + sulfasalazine-treated cells than in NAC and l-cysteine (*p* < 0.05 and *p* < 0.01, respectively; Fig. 5b). This increase in glutamate with NAC and l-cysteine treatment was attenuated when sulfasalazine was applied for Xc^−^ inhibition, indicating that Xc^−^ is involved in cysteine and NAC transport into the cell. Inhibition of Xc^−^ with sulfasalazine also prevented the increase in intracellular GSH seen with l-cysteine treatment, but this was not seen with NAC. This indicates that although Xc^−^ may be the primary mechanism of transport of cysteine and NAC intracellularly, it is possible that other mechanisms of NAC transport may become involved when Xc^−^ is inhibited, which may help increase cellular glutathione levels. However, the response to D-NAC treatment (a significant increase in glutathione without an increase in extracellular glutamate) was seen irrespective of sulfasalazine treatment, indicating that D-NAC bypasses this antiporter.

This would explain the increase in glutathione that is seen even when sulfasalazine was used to block Xc^−^. However, the glutamate level is significantly lower when sulfasalazine was used, indicating that the uptake mechanism may be one other than Xc^−^. In the absence of sulfasalazine, glutamate levels are higher for both NAC and l-cysteine, implying that the primary mechanism of transport may be through Xc^−^. However, the glutamate level was lower when sulfasalazine was used to block the Xc^−^.

#### D-Cy5 co-localizes in microglia and astrocytes in *Mecp2*-null mice but not in WT mice

**Fig. 6:**
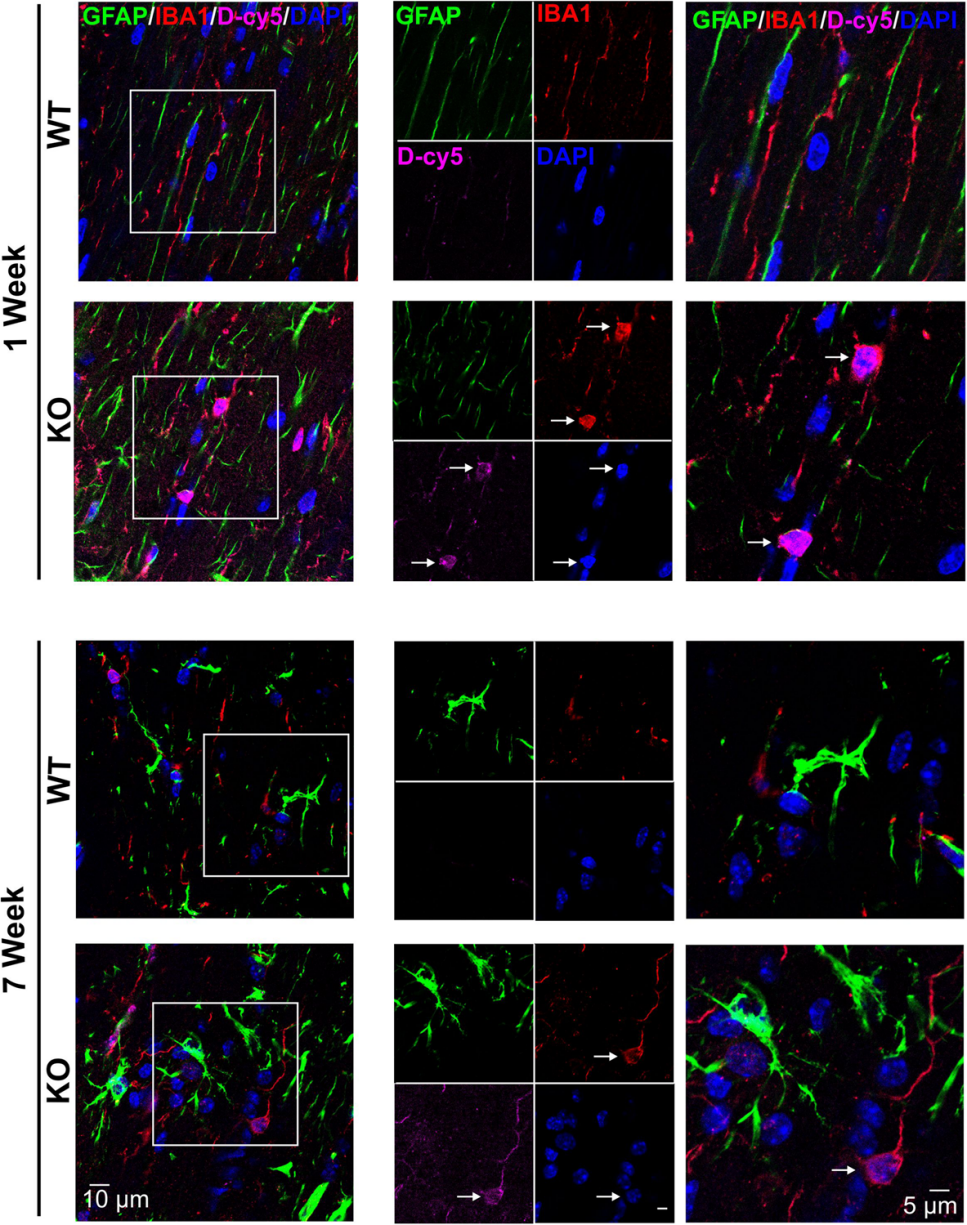
Brain uptake and cellular localization of D-Cy5 in WT and *Mecp2*-null mice. Dendrimer labeled with Cy5 (D-Cy5; magenta) was taken into microglia (Iba1, red) in the pre-symptomatic period (1 week of age) and in the symptomatic period (7 weeks of age). Little-to-no uptake of D-Cy5 was observed in astrocytes (GFAP; green). White arrows indicated D-Cy5 co-localization with Iba+ cells. Cell nuclei were stained with DAPI (blue). Scale bars = 10 or 5 μm

In the Bird RTT mouse model, *Mecp2*-null mice became phenotypic around 3 weeks of age and had a life expectancy of 6–10 weeks of age [53]. Therefore, we characterized dendrimer biodistribution using a dendrimer conjugated with the fluorescent tag, Cy5 (D-Cy5), in the pre-phenotypic period at 1 week of age and at 7 weeks of age when the symptoms were severe. All animals were administered 55 mg/kg of D-Cy5 and sacrificed and perfused with phosphate-buffered saline 24 h later. Microglia were visualized in fixed tissue using Iba1 staining, and astrocytes were visualized with GFAP staining. In WT mice, D-Cy5 uptake was rarely seen. In *Mecp2*-null mice, we found dendrimer uptake in microglia but not astrocytes at both 1 and 7 weeks of age (Fig. 6).

#### D-NAC treatment improved symptoms in *Mecp2*-null mice

**Fig. 7:**
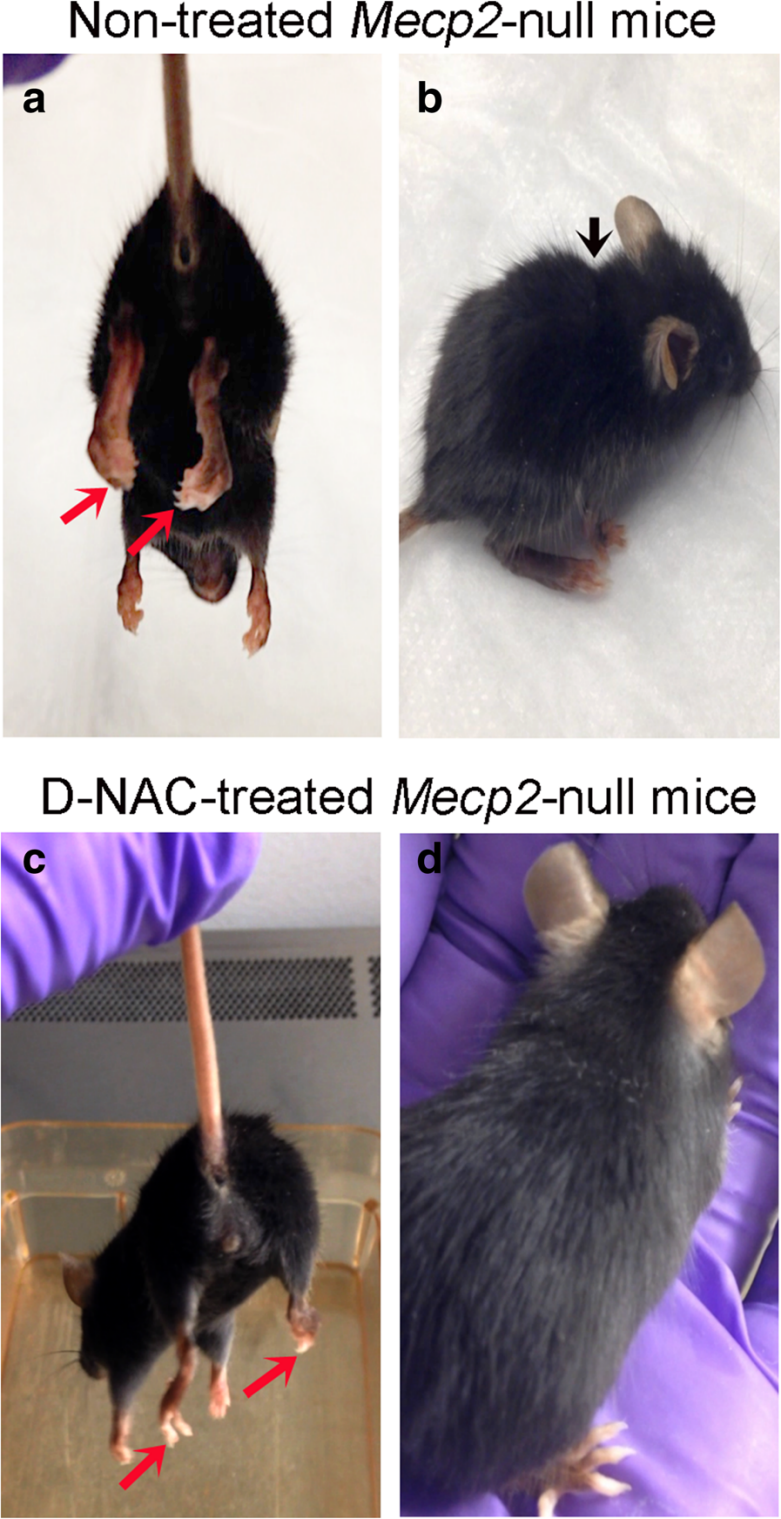
The physical appearance of *Mecp2*-null mice was improved by D-NAC therapy. Non-treated *Mecp2*-null mice were emaciated and had severe paw clenching, hunched posture, and poor eye conditions (**a**, **b**). D-NAC improved the overall appearance of *Mecp2*-null mice (**c**, **d**)



To evaluate the effect of an anti-oxidant/anti-inflammatory therapy that is targeted to activate microglia and astrocytes in this model, *Mecp2*-null and WT mice received intraperitoneal injections of D-NAC (10 mg/kg on a NAC basis), NAC (10 mg/kg), or PBS twice weekly starting at PD 21, the average age when animals become symptomatic in this model. Animals were weighed twice a week, and their behavior was assessed prior to treatment. The overall appearance of the *Mecp2*-null mice improved after D-NAC treatment. By 7 weeks of age, PBS-treated *Mecp2*-null mice were emaciated, hunched, were unable to groom, and had their hind paws clenched (Fig. 7a, b; Additional file 5). D-NAC-treated *Mecp2*-null mice maintained their appearance for a longer period and had less hind paw clenching (Fig. 7c, d; Additional file 6).

**Additional file 5:** *Mecp2*-null saline-treated mouse neurobehavior over time. Video of neurobehavioral testing over time demonstrating increase in phenotypic features (gait/waddle, paw clench, respiration abnormalities, and impairments in mobility). (MOV 55272 kb)


**Fig. 8:**
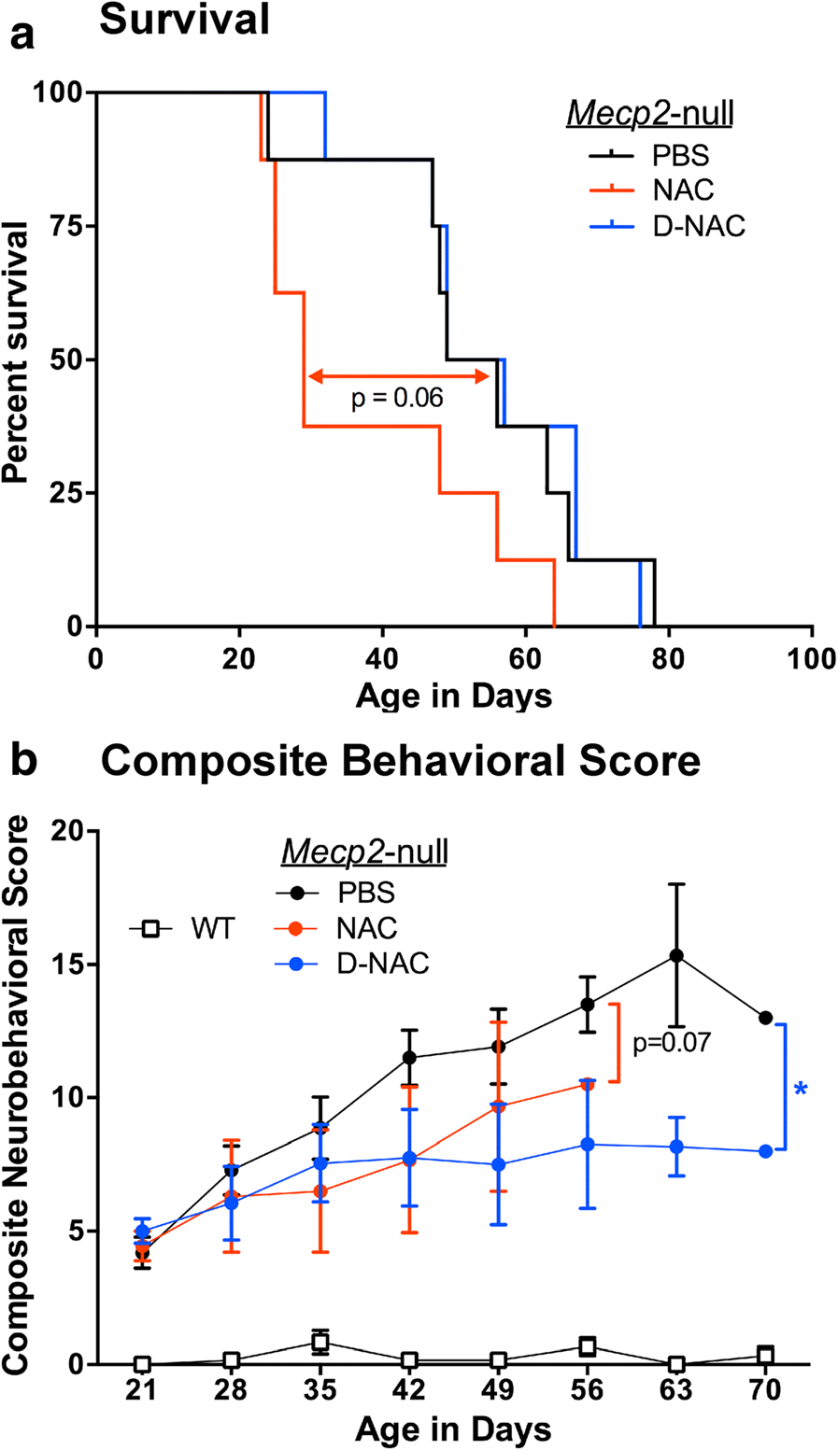
Effects of D-NAC on neurobehavioral outcomes and survival in *Mecp2*-null mice. **a** Twice weekly injections of 10 mg/kg doses of D-NAC treatment significantly improved the composite behavioral score compared to vehicle (PBS)-injected *Mecp2*-null mice (*p < 0.05). There was a trend for the composite behavioral score to be lower in NAC-treated *Mecp2*-null mice (*p* = 0.07). **b** Survival was assessed using a Kaplan-Meier curve. D-NAC did not improve survival compared to PBS-treated animals. D-NAC-treated *Mecp2*-null mice showed a trend for longer survival time (*p* = 0.06) compared to NAC-treated mice



Using a scoring system developed for the Bird model of *Mecp2* sufficiency [53], behavioral features that were scored included mobility, gait, tremors, paw clenching, clench time, paw wringing, and respiration, with each feature being scored on a scale of 0 to 3, with 0 being normal and 3 being severely affected (Additional file 1). Age-matched WT mice served as controls and showed an average score of 0 (healthy) on all behavioral tests. Higher composite behavioral scores indicated a more severe phenotype. *Mecp2*-null mice treated with D-NAC showed a significant improvement in behavior by PD 35 compared to PBS-treated *Mecp2*-null mice and continued to show a slowed progression of the phenotype (*p* < 0.05; Fig. 8a). *Mecp2*-null mice that were treated with PBS or NAC showed a steeper progression of symptoms over time (Fig. 8a; Additional file 5). NAC mice showed a trend for improvement in comparison to PBS-treated mice (*p* = 0.07; Fig. 8a). However, D-NAC did not significantly improve survival of *Mecp2*-null mice compared to the PBS-treated group (Fig. 8b). The 50% survival of both PBS-treated *Mecp2*-null pups and D-NAC-treated *Mecp2*-null pups was 49 PDs, 7 weeks of age. However, the 50% survival of NAC-treated mice was much lower at 25 days old, which approached significance (*p* = 0.06) compared to D-NAC and PBS-treated *Mecp2*-null mice. Likewise, NAC and D-NAC treatments did not alter the weight loss observed in *Mecp2*-null mice with age (Additional file 7).

**Additional file 6:** *Mecp2*-null D-NAC-treated mouse neurobehavior over time. Video of neurobehavioral testing over time demonstrating representative phenotype for this experimental group over time. D-NAC-treated mice show a slower increase in phenotypic severity as compared to saline-treated mice. (MOV 63483 kb)


## Discussion

Mutations in the *Mecp2* gene have been linked to both dysregulation/abnormal function of the immune system [8, 9, 11, 12, 54, 55] and dysregulation of redox homeostasis [56]. Patients with either *MECP2* or *CDKL5* mutations exhibit dysregulated cytokine expression and elevated erythrocyte sedimentation rate which may be indicative of chronic inflammation [8]. Increasing TNF-α levels also have been shown to correlate with worsening clinical severity in RTT, and patients with *CDKL5* mutations have also been shown to have higher levels of IL-10 [8]. A recent report described increased TNF-α and IL-6 expression along with decreased TGF-β expression in *Mecp2*-null mice brains suggestive of immune dysregulation and a shift to a pro-inflammatory state in the absence of *Mecp2* [9]. The changes were most pronounced in severely affected mice (average age 8–12 weeks). In that study, the authors described both a decrease in microglial numbers and a change in the microglial morphology to a more *activated* form in the severely symptomatic mice [9]. Our in vitro findings of increased baseline TNF-α and IL-10 levels in mixed glial cultures from *Mecp2*-null mice parallel these in vivo results. Exposure to LPS resulted in an exaggerated response of all the cytokines, indicating that the *Mecp2*-null glia were hyper-responsive to an inflammatory stimulus. IL-12 may mediate this response since levels were disproportionately increased by LPS stimulation. IL-12 has been shown to be critical in driving microglia to an M1 or pro-inflammatory phenotype [57–59]. Although LPS was used to stimulate the glia in vitro in this study, in the clinical setting, patients with Rett syndrome are known to have frequent respiratory infections that may be related to their scoliosis or presence of gastroesophageal reflux [60]. Exposure to infections resulting in systemic inflammation may activate these hyper-responsive glia in Rett syndrome patients worsening the underlying neurologic injury.

After LPS stimulation, we also found increased release of CXCL1, a chemokine shown to be involved in angiogenesis, nociception, and neutrophil recruitment [61, 62]. CXCL1, like other chemokines and cytokines, is a downstream target of NF-κB, which has been previously shown to be upregulated in a mouse model of RTT [63]. Other targets of NF-κB are upregulated in RTT, and it also has been demonstrated that decreasing NF-κB activity lowers the levels of these targets [63]. However, we did not see decreases in CXCL1 levels with D-NAC administration (upstream of NF-κB) in primary mixed glial cultures from *Mecp2*-null mice. It is possible that lengthening treatment and post-treatment phases could alter levels of transcription.

Microglia and macrophages express relatively low levels of *Mecp2* relative to neurons [10], but it has recently been shown that subsets of macrophage populations, including microglia, are particularly vulnerable to a loss of *Mecp2* early in the disease progression [9]. MECP2 appears to regulate the inflammatory response of microglia and macrophages, and *Mecp2* deletion leads to impaired responses by microglia/macrophages to stimuli such as hypoxia and inflammation [9]. The global loss of *Mecp2* also leads to reductions in the numbers of microglia and perivascular meningeal macrophages [9]. While the role of microglia in mediating RTT phenotype remains elusive, it is clear that the dysregulated inflammatory and phagocytic functions in microglia could lead to changes in normal brain circuitry and function [64].

A recent study showed that serum of patients with RTT exhibited an abnormal redox status with increases in markers of protein or lipid oxidative injury [8]. An increase in oxidative markers can drive the inflammation and further worsen it. In our studies, we demonstrated an increase in NO with LPS treatment but there was no evidence of differences between the groups at baseline or after LPS administration. Regardless of genotype, D-NAC was 10-fold more effective than NAC at reducing NO concentration. This aligns with previous in vitro and in vivo data of ours that demonstrates a 10–100-fold increase in efficacy of dendrimer-delivered NAC compared to free NAC [32].

Our findings of increased Xc^−^ expression in the brain tissue from *Mecp2*-null mice and in mixed glial cells after LPS stimulation in WT and *Mecp2*-null mice are consistent with others showing upregulation of Xc^−^ in microglia and macrophages in the presence of inflammation [65]. Upregulation of Xc^−^ also has been reported in several neurodegenerative and neuroinflammatory disorders such as amyotrophic lateral sclerosis (ALS) and Parkinson’s and Alzheimer’s diseases, and Xc^−^ has recently become a therapeutic target for these disorders. System Xc^−^ has been demonstrated to regulate microglial glutamate release and toxicity, and its inhibition leads to decreased neuronal and oligodendrocyte toxicity and slows symptoms in ALS and experimental autoimmune encephalomyelitis models of multiple sclerosis [66–68].

The rise in extracellular glutamate levels observed with free NAC and l-cysteine treatment was blocked when Xc^−^ was inhibited with sulfasalazine. Although NAC and l-cysteine are transported primarily into the cell by Xc^−^, other transporters such as the alanine-serine-cysteine (ASC) transporter may also be involved in the intracellular uptake of NAC and cysteine [69]. NAC may also utilize other pathways for entry intracellularly [18, 28, 70]. Inhibition of Xc^−^ led to a significant decrease in GSH for l-cysteine and a slight decrease for NAC that did not reach significance. However, the increase in glutamate seen with NAC and l-cysteine treatment was blunted in the presence of sulfasalazine, indicating that in the absence of Xc^−^ inhibition, NAC and cysteine may be primarily transported through this antiporter. We have previously shown that D-NAC is internalized into cells by an active endocytotic process [71] and hence bypasses the system Xc^−^. This allows for increases in intracellular glutathione without alterations in extracellular glutamate.

Some patients with RTT, as well as mouse models of RTT, exhibit increased glutamate levels in the CSF [72]. Furthermore, it has been shown that *Mecp2*-null microglia produce five times the normal amount of glutamate [10]. The results of previous studies of postmortem tissue and studies with the Bird mouse model of *Mecp2* insufficiency indicate that alterations in glutamate homeostasis and in the developmental expression of NMDA receptors (NMDARs) in the cortex may contribute to synaptic dysfunction in RTT [42, 73, 74]. The increased NMDARs both in the patients and in the mouse model with RTT at young ages were associated paradoxically with increased glutamate levels, indicating hyper-excitability and increased susceptibility to glutamate in these patients [42, 74, 75]. Thus, further increasing glutamate release with l-cysteine/NAC uptake through Xc^−^ transporter could have more deleterious consequences. In contrast, since D-NAC uptake bypasses system Xc^−^, we would not expect an increase in extracellular glutamate levels regardless of the concentration. This suggests that D-NAC can improve the safety and efficacy of NAC in this model. This may also explain the increased mortality and worse outcomes seen with free NAC treatment in this model.

Dendrimer is taken up selectively by cells involved in inflammation in several models of brain injury, with specific cell localization dependent on the disease model and the time of dendrimer administration after injury [32–34]. The mechanism of cellular uptake of dendrimers is shown to be dependent on its surface charge and size and is mediated predominantly by endocytosis and/or micropinocytosis [76]. We used generation-4 hydroxyl-terminated PAMAM dendrimers that were of neutral charge and about 4 nm in size. We previously demonstrated that these dendrimers were taken up by fluid-phase endocytosis [52]. In this study, we found that D-NAC bypassed the Xc^−^ antiporter to transport NAC into the cell. This avoids the extracellular glutamate release by the antiporter, thereby preventing further injury in conditions such as RTT where excess glutamate and associated excitotoxicity is commonly described [77–79].

This study is the first to look at dendrimer distribution and localization as a function of both genotype and age in a mouse model of RTT. Although altered phagocytosis has been described in *Mecp2*-null microglia previously, this does not affect dendrimer uptake by the cells because neutral-charged dendrimers are predominantly taken up by endocytotic mechanisms [52, 80, 81].

The improved efficacy of D-NAC over free NAC may be due to the selective targeting and increased uptake of dendrimers into activated glia, leading to a higher drug concentration specifically in these cells. The in vitro studies with primary mixed glial cells were designed to mimic the clinical scenario where drug exposure to the cell occurs for a short time before it gets cleared from the circulation. D-NAC accumulates and is retained in the activated glia, releasing the drug over time even after it is removed from the supernatant. Since every dendrimer has a payload of ~ 20 molecules of NAC, a larger amount of the drug is delivered to the cells when compared to free NAC. In vivo pharmacokinetic studies with NAC have shown poor accumulation in the brain and spinal cord, with limited ability of NAC to cross the BBB [69, 82]. Although this may increase in the presence of an impaired blood-brain barrier, dendrimer enables specific cellular targeting of NAC to microglial cells. In the in vivo setting, NAC increases GSH levels primarily by displacement of plasma protein-bound cysteine [83]. Cysteine and its dimer cystine are then transported into the brain across the BBB and intracellularly to increase cellular GSH levels [83]. D-NAC is designed and synthesized so that it does not release the drug in extracellular conditions and protects early metabolism of NAC prior to cellular internalization (please see release data provided) [32]. Although D-NAC was effective in delaying symptom progression, it did not improve survival compared to PBS-treated *Mecp2*-null mice. This indicates that other combination therapies may be required along with D-NAC to enhance the efficacy.

Our lab has previously demonstrated that hydroxyl-terminated PAMAM dendrimers can target activated microglia (and astrocytes) in several models of brain injury mediated by neuroinflammation [32–34]. Therefore, the ability of dendrimers to localize in microglia in the brain of *Mecp2*-null mice could have significant therapeutic implications. We have previously shown that D-NAC is more effective than a 10–100 times higher dose of NAC in improving motor deficits, myelination, and neuronal counts and in decreasing inflammation and microglial activation in a rabbit model of CP [32]. In this study, we demonstrated that D-NAC, given systemically twice weekly, resulted in an improvement in overall neurobehavioral score and in microglial morphology compared to PBS-treated *Mecp2*-null mice. D-NAC was significantly more effective in decreasing inflammatory cytokines and oxidative stress and in improving intracellular GSH levels at the lowest doses when compared to free NAC even at the highest doses, in the in vitro studies.

Several pre-clinical studies have implicated microglia and astrocytes as therapeutic targets in the treatment of RTT. The dysregulation in immune homeostasis in *Mecp2*-null mice described by this study and others [9, 14] may indicate that impaired glial function and its inability to maintain immune homeostasis may affect normal brain development and response to injury in RTT. The dendrimer platform offers such an opportunity to specifically manipulate the glial responses in this challenging disorder. Therapeutics targeting glutamate inhibition, GABA and NMDA inhibitors, or delivery of BDNF in combination with a dendrimer platform that can target activated glia may have the potential for reversing phenotypes in RTT, improving neurological outcomes, and increasing survival. Future studies will include evaluation of D-NAC therapy in *Mecp2*-heterozygous mice and more quantitative measures of behavioral outcomes and respiration.

## Conclusion

This study demonstrated dysregulated microglia and neuroinflammatory processes in a mouse model of RTT. *Mecp2*-null glial cells and brain tissue showed increased expression of Xc^−^ mRNA, suggesting increased sensitivity to oxidative stress. Furthermore, *Mecp2*-null glial cell cultures exhibited an exaggerated response to LPS stimulation compared to WT glial cell cultures indicative of immune dysregulation that was improved after the targeted delivery of an anti-inflammatory/anti-oxidant agent to glial cells using dendrimer-based delivery of NAC. D-NAC also was effective in slowing the progression of the RTT phenotype in *Mecp2*-null mice most likely by bypassing Xc^−^, a possible mechanism for toxicity seen with NAC administration. Further studies are needed to improve efficacy of D-NAC alone or in combination with other therapies in animal models of RTT. Dendrimer-mediated delivery of therapies that target neuroinflammation and oxidative stress, as well as other neuropathological processes observed in RTT, including glutamate and GABA dysregulation, could advance treatment for RTT symptoms and improve quality of life and survival in patients with RTT.

## Additional files

Additional file 1: Neurobehavioral Scoring Scheme. Table of neurobehavioral subscores and their scoring scheme used to determine phenotypic severity in *Mecp2*-null mice. (PDF 40 kb)

Additional file 2: Physiochemical characterization of dendrimer-N-acetylcysteine (D-NAC) conjugates. A) D-NAC chemical structure and proton NMR spectrum of D-NAC conjugate in DMSO. B) HPLC chromatogram of D-NAC with elution time at 19.65 min and free NAC eluting at 6.14 min. C) Size and zeta potential measurements of G4-OH, bifunctional dendrimer and D-NAC conjugates. (TIFF 2366 kb)

Additional file 3: Evaluation of NAC release from D-NAC was conducted in human pooled plasma. (A) To simulate an extracellular environment, 10 μM of GSH was added to the plasma containing D-NAC (3 mg/mL). D-NAC was stable in plasma for more than 48 h releasing ~ 5% of its payload. (B) For intracellular stimulation, 250 μM of GSH was used. D-NAC demonstrated faster NAC release (~ 80% of its payload) within 5 h suggesting that GSH cleaved the disulfide bonds. (JPEG 43 kb)

Additional file 4: MTT cytotoxicity assay. No toxicity of G4-OH dendrimer, NAC, or dendrimer-conjugated NAC was observed in *Mecp2*-null primary mixed glial culture at 100 and 10 μg/ml concentrations. There was some toxicity with free NAC at 200 μg/ml (< 80% cell viability). Therefore, we did not use this concentration in any further experiments. (TIFF 213 kb)

Additional file 7: D-NAC treatment did not prevent weight loss in Mecp2-null mice. Regression plots for body weights of WT (open squares-dotted line) and Mecp2-null mice treated with PBS (solid black circles-black line), NAC (red open circles-red line) and D-NAC (solid blue circles-blue line). With age, weight increased in all groups of mice. However the trajectory for all groups of Mecp2-null mice was less than in WT mice. (TIFF 236 kb)

## Abbreviations

BBB: Blood-brain barrier
BDNF: Brain-derived neurotrophic factor
CXCL1: C-X-C motif chemokine 1
D-NAC: Dendrimer-conjugated *N*-acetyl cysteine
GABA: Gamma-aminobutyric acid
GSH: Glutathione
GSSG: Glutathione disulfide
h: Hours
IL-2: Interleukin-2
IL-4: Interleukin-4
IL-5: Interleukin-5
IL-6: Interleukin-6
IL-10: Interleukin-10
IL-12p70: Interleukin-12
LPS: Lipopolysaccharide
*MECP2*: Methyl-CpG-binding protein 2 human gene
*Mecp2*: Methyl-CpG-binding protein 2 mouse gene
min: Minutes
NAC: *N*-Acetyl cysteine
NMDA: *N*-Methyl-d-aspartate
PAMAM: (Poly)amidoamine
PD: Postnatal day
RTT: Rett syndrome
TGF-β: Tumor growth factor beta
TNF-α: Tumor necrosis factor alpha
Xc^−^: Cysteine-glutamate antiporter
