# Correction to: Dendrimer-mediated delivery of N-acetyl cysteine to microglia in a mouse model of Rett syndrome

**DOI:** 10.1186/s12974-018-1056-1

**Published:** 2018-01-12

**Authors:** Elizabeth Nance, Siva P. Kambhampati, Elizabeth S. Smith, Zhi Zhang, Fan Zhang, Sarabdeep Singh, Michael V. Johnston, Rangaramanujam M. Kannan, Mary E. Blue, Sujatha Kannan

**Affiliations:** 1 Department of Anesthesiology and Critical Care Medicine, Johns Hopkins University School of Medicine, Baltimore, MD 21205 USA; 2 Center for Nanomedicine, Department of Ophthalmology, Wilmer Eye Institute, Johns Hopkins University School of Medicine, Baltimore, MD 21231 USA; 3 Department of Chemical and Biomolecular Engineering, Johns Hopkins University, Baltimore, MD 21218 USA; 4 Department of Materials Science and Engineering, Johns Hopkins University, Baltimore, MD 21218 USA; 5 Hugo W. Moser Research Institute, Kennedy Krieger, Inc, Baltimore, MD 21205 USA; 6 Present address: Department of Chemical Engineering, University of Washington, Seattle, WA 98105 USA

## Correction

After publication of the article [1], it has been brought to our attention that an author’s name has been formatted incorrectly. The correct name should be “Rangaramanujam M. Kannan” but it was previously included as “Kannan Rangaramanujam”. The original version has now been revised to reflect this.
