# Supplementary material for: Dendrimer-mediated delivery of N-acetyl cysteine to microglia in a mouse model of Rett syndrome
Source: J Neuroinflammation. 2017 Dec 19;14:252. doi: 10.1186/s12974-017-1004-5 (PMC5735803; doi:10.1186/s12974-017-1004-5)
Supplement: Supplementary file 3 — Neurobehavioral Scoring Scheme. Table of neurobehavioral subscores and their scoring scheme used to determine phenotypic severity in Mecp2-null mice. (PDF 40 kb) [file 12974_2017_1004_MOESM1_ESM.pdf]

Subscale and score	Criteria
Mobility	
0	normal, active
1	decreased spontaneous movement
2	no spontaneous movement, only moves when prodded
3	no movement
Gait	
0	walks normally
1	waddling
2	waddling, walks on toes (paws not flat on ground)
3	waddling, feet displaced outward, paws clenched when walking
Tremors	
0	none
1	Sporadic
2	Intermittent
3	Continuous
Paw Clenching	
0	none
1	1-2 paws
2	3 paws or both fore limbs/hind limbs
3	all paws
Clench time	
0	none
1	sporadic
2	intermittent (patterned)
3	continuous
Paw wringing	
0	none
1	some front paw
2	some front and back paw
3	continuous
Respiration	
0	regular
1	irregular
2	too fast or too slow
3	apnic
