# Supplementary figures and images for: Dendrimer-mediated delivery of N-acetyl cysteine to microglia in a mouse model of Rett syndrome
Source: J Neuroinflammation. 2017 Dec 19;14:252. doi: 10.1186/s12974-017-1004-5 (PMC5735803; doi:10.1186/s12974-017-1004-5)

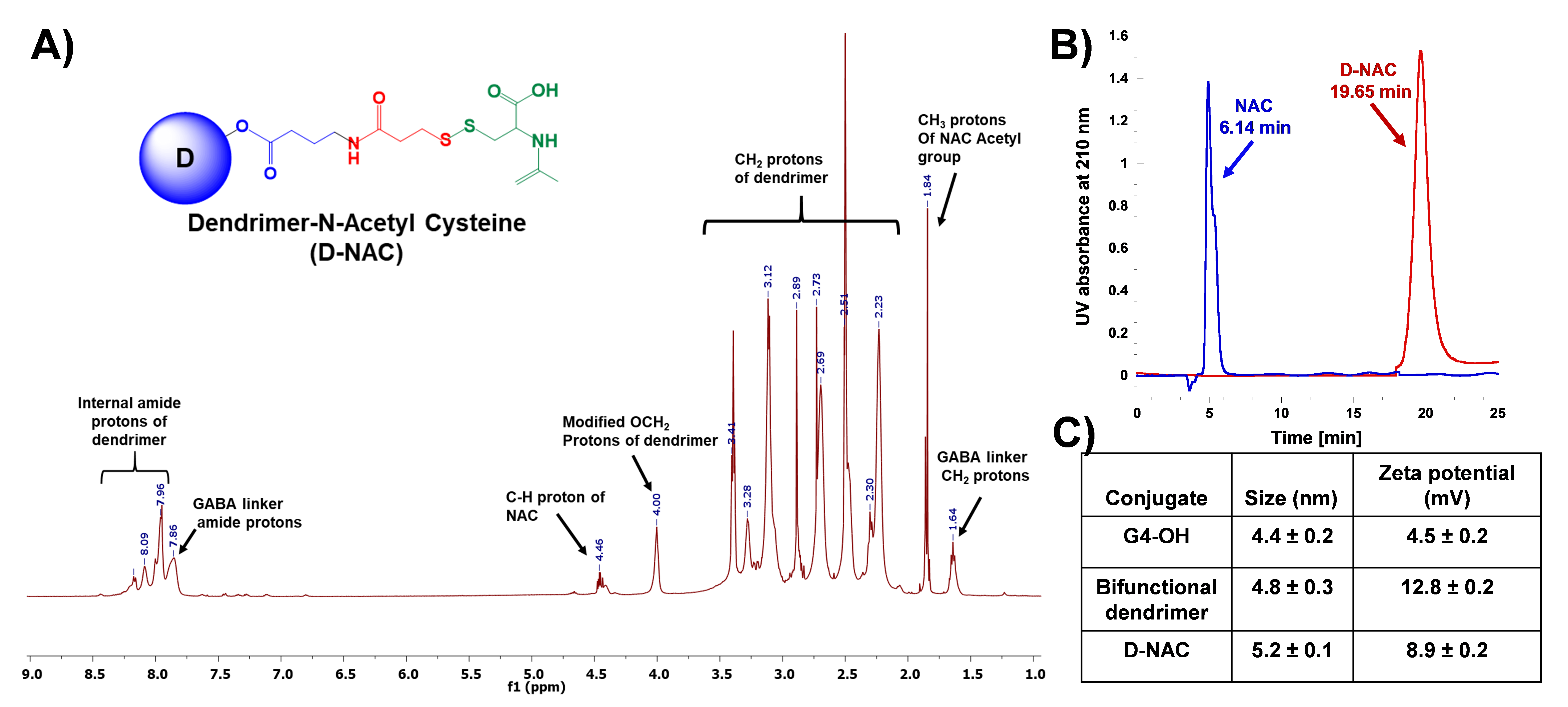

Supplement: Supplementary file 4 — Physiochemical characterization of dendrimer-N-acetylcysteine (D-NAC) conjugates. A) D-NAC chemical structure and proton NMR spectrum of D-NAC conjugate in DMSO. B) HPLC chromatogram of D-NAC with elution time at 19.65 min and free NAC eluting at 6.14 min. C) Size and zeta potential measurements of G4-OH, bifunctional dendrimer and D-NAC conjugates. (TIFF 2366 kb) [file 12974_2017_1004_MOESM2_ESM.tif]

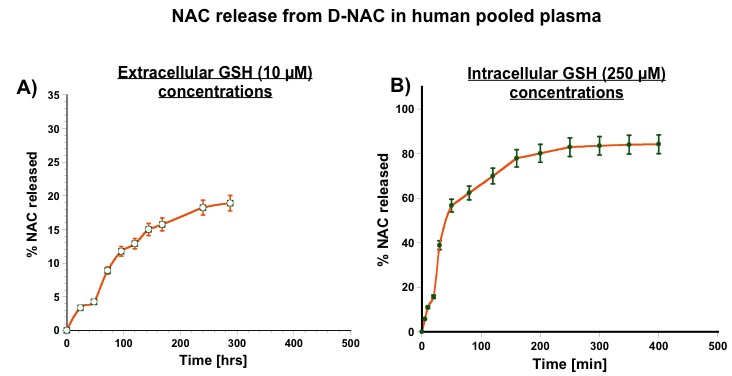

Supplement: Supplementary file 5 — Evaluation of NAC release from D-NAC was conducted in human pooled plasma. (A) To simulate an extracellular environment, 10 μM of GSH was added to the plasma containing D-NAC (3 mg/mL). D-NAC was stable in plasma for more than 48 h releasing ~ 5% of its payload. (B) For intracellular stimulation, 250 μM of GSH was used. D-NAC demonstrated faster NAC release (~ 80% of its payload) within 5 h suggesting that GSH cleaved the disulfide bonds. (JPEG 43 kb) [file 12974_2017_1004_MOESM3_ESM.jpg]

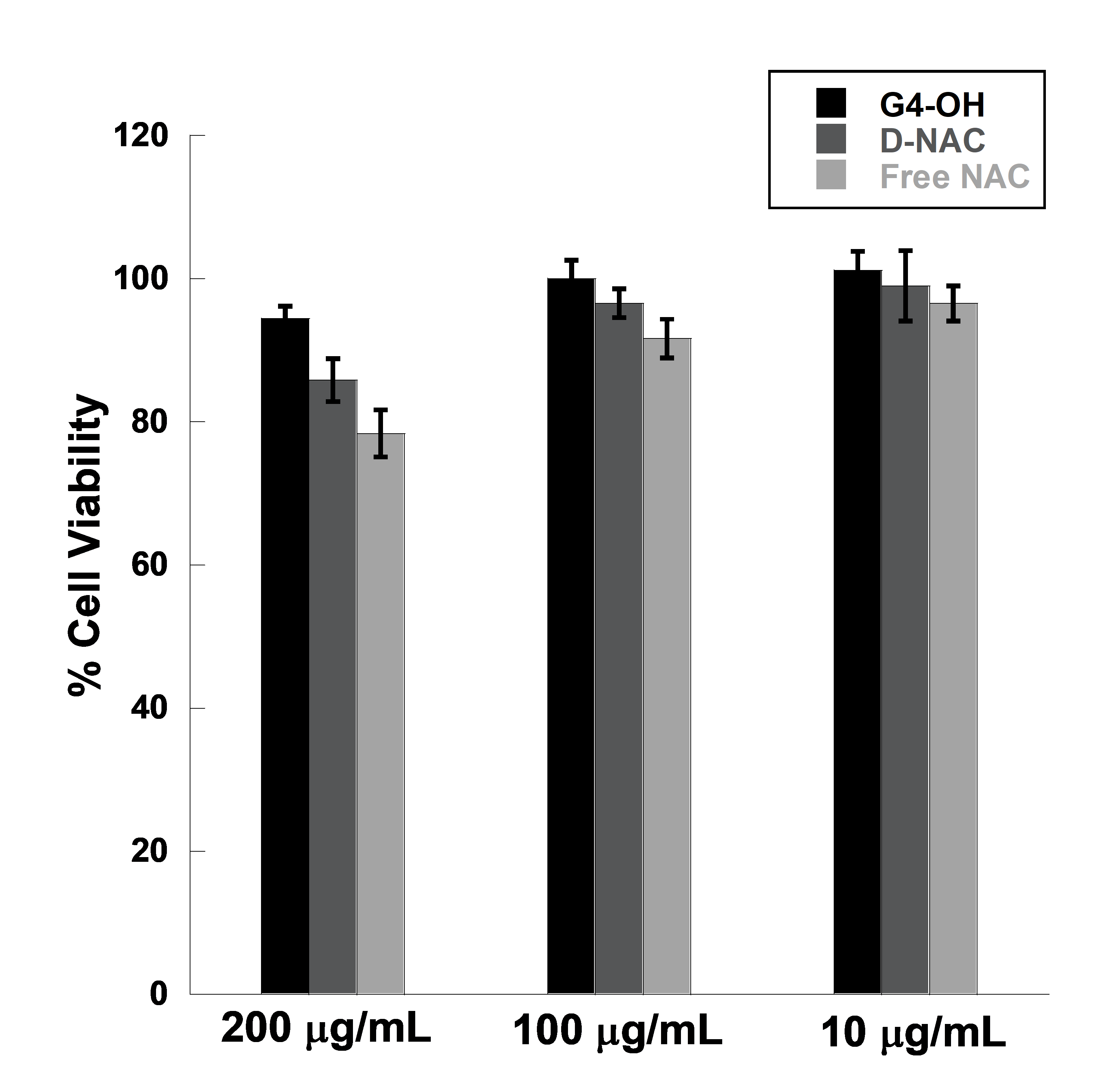

Supplement: Supplementary file 6 — MTT cytotoxicity assay. No toxicity of G4-OH dendrimer, NAC, or dendrimer-conjugated NAC was observed in Mecp2-null primary mixed glial culture at 100 and 10 μg/ml concentrations. There was some toxicity with free NAC at 200 μg/ml (< 80% cell viability). Therefore, we did not use this concentration in any further experiments. (TIFF 213 kb) [file 12974_2017_1004_MOESM4_ESM.tif]
